# Intersegmental Modulation of Lower‐Limb Corticospinal Excitability and Inhibition Induced by Upper‐Limb Isometric Contractions: A Systematic Review

**DOI:** 10.1111/ejn.70501

**Published:** 2026-04-12

**Authors:** Gustavo Henrique de Mello Rosa, Gabrielly Santos Pereira, Fernanda da Silva Boni, Marcelo Lourenço da Silva, João Eduardo de Araújo

**Affiliations:** ^1^ Neuropsychobiology and Motor Control Laboratory, Department of Health Sciences, Ribeirão Preto Medical School University of São Paulo São Paulo Brazil; ^2^ Laboratory of Neuroscience, Neuromodulation and Study of Pain (LANNED) Federal University of Alfenas (UNIFAL‐MG) Alfenas Brazil

**Keywords:** isometric contraction, motor cortex, neural inhibition, pyramidal tracts, transcranial magnetic stimulation

## Abstract

Intersegmental neural modulation refers to the influence of voluntary activation of one limb on the excitability and inhibitory balance of remote motor representations. Recent evidence suggests that high‐intensity upper‐limb isometric contractions can transiently enhance corticospinal excitability and modulate intracortical or interhemispheric inhibition of lower‐limb motor areas, yet the consistency of these findings remains unclear. This systematic review synthesized evidence on the effects of upper‐limb isometric contractions on corticospinal excitability and inhibitory mechanisms of lower‐limb motor representations in healthy adults. Following PRISMA 2020 guidelines, searches were conducted in PubMed/MEDLINE, Scopus, Web of Science, Embase, and Cochrane CENTRAL. Eligible studies included healthy adults (18–65 years) performing upper‐limb isometric contractions quantified as percentage maximal voluntary contraction (%MVC), with outcomes assessing lower‐limb corticospinal excitability or intracortical/interhemispheric inhibition. Two independent reviewers screened studies, extracted data, and assessed risk of bias. Seventeen studies (*n* = 351) met inclusion criteria. Most reported increases in lower‐limb motor evoked potential amplitude during upper‐limb contractions, particularly at intensities ≥ 70% MVC. Reductions in short‐interval intracortical inhibition were common, indicating transient disinhibition of lower‐limb primary motor cortex representations. Findings for interhemispheric inhibition were inconsistent, likely attributable to variability in contraction tasks and transcranial magnetic stimulation parameters. Upper‐limb isometric contractions consistently facilitate lower‐limb corticospinal excitability and reduce intracortical inhibition in healthy adults. Although mechanistic patterns converge, methodological heterogeneity limits confidence in the magnitude of effects. Future studies require standardized experimental protocols and adequate sample sizes to clarify intensity–response relationships and underlying neurophysiological mechanisms.

## Graphical abstract

Moderate‐intensity isometric contractions in the upper limbs (10%–30% MVC) facilitate corticospinal excitability of motor representations of the lower limbs, while high‐intensity efforts (exceeding 70% MVC) shift modulation toward inhibition. The effect is mediated by supraspinal intersegmental networks and is characterized by increased motor evoked potentials and reduced intracortical inhibition in the muscles of the lower limbs.

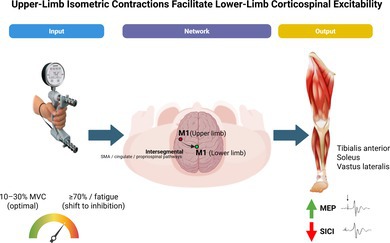

**Abbreviations**
ADM: abductor digiti minimi
BB: biceps brachii
BFO: blood flow occlusion
BR: brachioradialis
CSP: cortical silent period
ECR: extensor carpi radialis
EMG: electromyography
ES: erector spinae
FDI: first dorsal interosseous
FCR: flexor carpi radialis
FDS: flexor digitorum superficialis
H‐reflex: Hoffmann reflex
ICF: intracortical facilitation
iSP: ipsilateral silent period
LAI: long‐latency afferent inhibition
LEP: laser‐evoked potential
LL: lower limb
M1: primary motor cortex
MEP: motor evoked potential
MVC: maximal voluntary contraction
PAS: paired associative stimulation
RA: rectus abdominis
RF: rectus femoris
SICI: short‐interval intracortical inhibition
SAI: short‐latency afferent inhibition
SEP: somatosensory evoked potential
SMA: supplementary motor area
SOL: soleus
SP: silent period
TA: tibialis anterior
TMS: transcranial magnetic stimulation
UL: upper limb
VL: vastus lateralis

## 1 Introduction

Human motor control emerges from the continuous interaction of cortical, subcortical, and spinal circuits that coordinate movement across the body (Carroll et al. 2006). Motor cortices operate within distributed networks rather than as isolated, effector‐specific modules, integrating representations across the primary motor cortex (M1), supplementary motor area (SMA), premotor regions, and propriospinal interneurons (Colomer‐Poveda et al. 2019). Foundational work in motor neuroscience has established core principles of interlimb coordination, indicating that coordinated behavior emerges from interactions within distributed neural networks rather than from fully independent, effector‐specific modules; this framework helps explain how the motor system integrates sensorimotor information to support complex behaviors such as locomotion and bimanual actions (Swinnen 2002; Swinnen and Carson 2002).

Neurophysiological and imaging studies demonstrate that activating one limb can modulate the excitability of remote muscles, reflecting intersegmental modulation and, under specific high‐demand conditions, related phenomena such as motor overflow (Tazoe and Komiyama 2014; Arya and Pandian 2014). Neuroimaging evidence further supports a distributed architecture involving SMA, cingulate motor regions, premotor and sensorimotor cortices, and the cerebellum during coordinated limb actions (Debaere et al. 2001; Gordon et al. 2023). This integrative coupling plays an essential role in locomotion, balance control, and bilateral motor tasks.

Although the terms motor overflow and intersegmental modulation are sometimes used interchangeably in the literature, they reflect partially distinct neurophysiological constructs. Motor overflow classically refers to the involuntary spread of motor activity to muscles not directly involved in the intended task, typically observed during high‐force or highly demanding contractions (Hoy et al. 2004; de Mello Rosa et al. 2025). In contrast, intersegmental modulation encompasses a broader and more controlled phenomenon, describing systematic changes in corticospinal excitability and inhibitory balance in remote motor representations induced by voluntary activation of a different limb or segment (McIntyre‐Robinson and Byblow 2013; Baldissera et al. 2002).

This modulation may occur even in the absence of overt electromyographic activity in the nonengaged muscles and reflects distributed interactions between supraspinal and spinal networks. Early paired‐coil TMS work described interhemispheric inhibitory interactions between motor cortices, supporting the role of transcallosal pathways in shaping motor output beyond the directly engaged representation (Ferbert et al. 1992). Subsequent studies extended this framework to nonhomologous interlimb interactions, showing that voluntary activation of one limb segment can modulate corticospinal excitability and inhibitory balance in distant motor representations (Kato et al. 2022). In this review, intersegmental modulation refers specifically to upper‐limb–induced changes in lower‐limb corticospinal and inhibitory circuits, whereas motor overflow is treated as a related but nonsynonymous phenomenon.

Early electromyographic and transcranial magnetic stimulation (TMS) studies revealed that unilateral upper‐limb contractions elicit measurable changes in contralateral and sometimes ipsilateral nonhomologous muscles (Giovannelli et al. 2009; Tazoe et al. 2007). These findings suggest that cross‐limb interactions may involve corticospinal output, transcallosal pathways, and contributions beyond the primary cortical representation. Functional MRI studies reinforce this interpretation by demonstrating engagement of SMA and cingulate motor areas during voluntary movement of a single limb (Hanakawa et al. 2003).

TMS provides a precise method to probe these multisite motor networks. Measures such as motor‐evoked potentials (MEPs), short‐interval intracortical inhibition (SICI), intracortical facilitation (ICF), and the ipsilateral silent period (iSP) allow detailed evaluation of how the nervous system regulates corticospinal excitability and intracortical or interhemispheric inhibition. Increased MEP amplitude reflects enhanced net corticospinal responsiveness, while reduced SICI indicates transient intracortical disinhibition, although MEPs may also include subcortical and spinal contributions (Kujirai et al. 1993; Rossini et al. 2015).

A growing body of evidence indicates that upper‐limb effort can modulate excitability in motor pathways controlling the lower limb. Early TMS experiments showed that unilateral hand contractions increase lower‐limb MEP amplitudes even when the leg remains at rest (Stinear and Byblow 2004; Perez et al. 2004). Subsequent studies using sustained or rhythmic arm tasks confirmed this cross‐limb facilitation; for example, rhythmic arm cycling increases corticospinal responsiveness and lower‐limb MEP amplitudes to the quadriceps and tibialis anterior (Loadman and Zehr 2007; Dragert and Zehr 2011). The magnitude of this facilitation scales with contraction intensity and task demands, suggesting a global modulation of descending motor output (Zehr and Duysens 2004; Christensen et al. 2000).

Cross‐limb effects also extend to inhibitory circuits. Paired‐pulse TMS studies often report reductions in SICI during remote muscle activation, suggesting a transient release of intracortical inhibition in nonengaged motor regions; for example, an isometric hand task reduces SICI in distant limb representations (Sohn and Hallett 2004). In contrast, high‐intensity or sustained contractions may enhance inhibitory indices, including longer cortical silent periods and larger iSPs, reflecting stronger interhemispheric inhibition under demanding conditions (Giovannelli et al. 2009; Gueugneau et al. 2017; Paish et al. 2023). These findings suggest that inhibitory control is dynamically regulated by task intensity and fatigue.

Although there is consistent evidence for cross‐limb influences, the literature is highly heterogeneous. Variations in contraction intensity, muscle selection, fatigue state, and TMS methodological parameters likely contribute to divergent findings. Furthermore, most research has concentrated on upper‐limb interactions or homologous muscle groups, with relatively few studies investigating upper‐to‐lower‐limb modulation. This gap limits understanding of whole‐body neural integration.

This gap has important translational relevance. If upper‐limb contractions reliably facilitate lower‐limb excitability, clinicians may leverage this effect to alter network excitability in individuals with stroke, spinal cord injury, or limb immobilization. Remote contractions may be conceptualized as a rehabilitation‐oriented priming strategy that could influence motor network state and responsiveness to training in conditions such as stroke, spinal cord injury, or immobilization (Stinear 2010). These possibilities underscore the need to consolidate existing evidence and clarify how intersegmental modulation can be harnessed in neurorehabilitation.

Although evidence describing intersegmental interactions is expanding, findings remain highly heterogeneous, and to date the literature has not been systematically synthesized with a specific focus on how upper‐limb isometric contractions modulate corticospinal excitability and inhibitory circuits in lower‐limb representations. Accordingly, a systematic synthesis is needed to consolidate evidence on how upper‐limb isometric contractions modulate lower‐limb corticospinal excitability and inhibitory circuits, clarify the role of contraction intensity and fatigue, and identify the supraspinal and spinal mechanisms involved. Such synthesis is essential to guide future research and support clinically meaningful neurorehabilitation strategies.

## 2 Methods

### 2.1 Study Design

This systematic review examined the intersegmental modulation of lower‐limb corticospinal excitability and inhibitory circuits induced by voluntary upper‐limb isometric contractions across different contraction intensities. The present review specifically focused on neurophysiological intersegmental modulation, defined as task‐dependent changes in remote motor representations that may occur even in the absence of overt electromyographic overflow. The review followed the PRISMA 2020 guidelines and was prospectively registered in PROSPERO (CRD420251183924).

### 2.2 Eligibility Criteria

Eligibility criteria were defined according to the PICOS framework:

- Population: Healthy adults aged 18–65 years without neurological, musculoskeletal, or psychiatric disorders. Studies involving clinical populations (e.g., stroke, spinal cord injury), pharmacological interventions, or animal models were excluded.
- Intervention: Studies investigating voluntary, sustained, rhythmic, or fatigue‐induced isometric contractions of upper‐limb muscles (hand, wrist, elbow, or shoulder). The primary focus of this review was on modulation recorded in lower‐limb motor representations. In addition, experimental studies examining intersegmental or interhemispheric modulation in other remote segments (e.g., trunk or contralateral upper limb) were included when they provided mechanistic insight relevant to the interpretation of lower‐limb outcomes.
- Comparison: Baseline or rest conditions, contralateral or ipsilateral limb comparison, or alternative contraction intensities (e.g., 10%–30% vs. 50%–75% MVC).
- Primary Outcomes: Neurophysiological modulation of lower‐limb motor representations, assessed through MEPs, intracortical inhibition/facilitation (SICI and ICF), or interhemispheric inhibition (iSP) elicited during upper‐limb activation.
- Secondary Outcomes: Studies that recorded modulation in remote nonlower‐limb muscles (e.g., contralateral hand, trunk muscles) or that assessed cortical/spinal inhibitory circuits (CSP, H‐reflex, reciprocal inhibition) were included as mechanistic evidence when they contributed to understanding the broader intersegmental regulation underlying upper‐to‐lower‐limb interactions.
- Study Design: Experimental studies assessing lower‐limb modulation constituted the primary dataset. Studies evaluating remote modulation in other segments were also included as secondary mechanistic evidence when relevant to the interpretation of intersegmental pathways.

Although the primary focus of this review was on studies directly assessing lower‐limb motor representations during upper‐limb isometric contractions, experimental studies examining intersegmental or interhemispheric modulation in other remote segments were included as secondary mechanistic evidence when they provided relevant insight into the cortical and spinal pathways underlying upper‐to‐lower‐limb interactions. These studies were not considered part of the primary evidence base but were used to support the interpretation of neurophysiological mechanisms.

### 2.3 Information Sources

Comprehensive electronic searches were conducted in PubMed, Scopus, Embase, and LILACS. Searches covered January 2007 to November 2025, with no language restrictions. Reference lists from eligible studies and previous reviews were hand‐searched to identify additional citations.

The search period was restricted to studies published from 2007 onward to capture investigations conducted after the consolidation of modern TMS methodologies, including standardized paired‐pulse paradigms for intracortical inhibition and facilitation, improved reporting of stimulation parameters, and the broader adoption of consensus guidelines for noninvasive brain stimulation. Earlier studies were screened through reference list searches to ensure that seminal work was not overlooked.

### 2.4 Search Strategy

A comprehensive and reproducible search strategy was developed in consultation with a research librarian. Controlled vocabulary terms (MeSH and Emtree) and free‐text keywords were combined using Boolean operators. The complete database‐specific syntax is provided in Supplementary Appendix S1.

### 2.5 Study Selection

**FIGURE 1:**
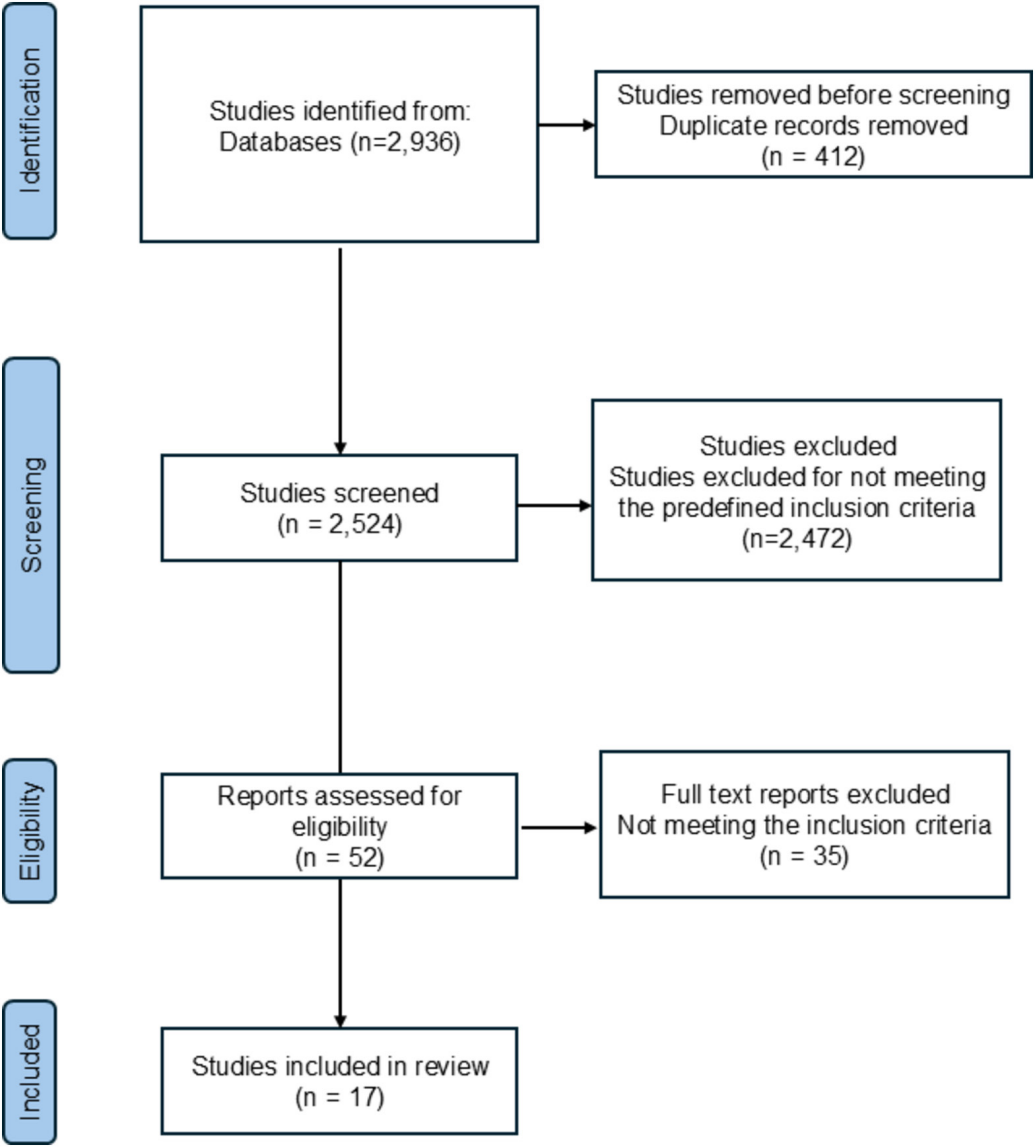
Flowchart of the review following the PRISMA guidelines.

All records were imported into Rayyan for duplicate removal and screening. Two independent reviewers (M.L.S. and G.S.P.) screened titles/abstracts and subsequently full‐texts. Disagreements were resolved through discussion or by a third‐party adjudicator (G.H.M.R.). The selection process is depicted in the PRISMA 2020 flow diagram (Figure 1).

### 2.6 Data Extraction

Two reviewers independently (M.L.S. and G.S.P.) extracted data using a standardized form, including: study identification (author, year, country, and journal); Sample size and demographics; contraction characteristics (%MVC, duration, and task); TMS parameters (muscle tested, hotspot, coil orientation, and intensity); neurophysiological outcomes (MEP, SICI, ICF, CSP, and iSP); main findings (direction and magnitude of modulation); and statistical significance; methodological features relevant to bias (blinding and order randomization). Study authors were contacted when essential data were missing or unclear.

### 2.7 Risk of Bias Assessment

Risk of bias was evaluated using the RoB 2.0 for randomized/crossover experimental designs and JBI critical appraisal tools for repeated‐measures laboratory studies. Each study was rated as low risk, some concerns, or high risk. The results were visualized using ROBVIS.

### 2.8 Data Synthesis

Given the heterogeneity in contraction tasks, intensities, TMS protocols, and outcome units, a qualitative (narrative) synthesis was conducted. Substantial heterogeneity was observed in stimulation intensity (90%–130% of resting motor threshold), coil orientation, and hotspot localization procedures across studies. Studies were grouped by the primary physiological domain examined: corticospinal excitability modulation (MEP amplitude changes); intracortical inhibition and facilitation (SICI, ICF, CSP); interhemispheric and spinal modulation (iSP, RI, afferent inhibition); intensity‐, duration‐, and fatigue‐dependent effects.

A narrative synthesis summarized trends in facilitation versus inhibition, direction and magnitude of effects, and consistency across studies.

A quantitative meta‐analysis was not performed due to substantial methodological heterogeneity across studies. Specifically, there was marked variability in contraction protocols (muscle group, intensity, duration, and fatigue state), outcome measures (raw vs. normalized MEP amplitude and timing of TMS delivery), stimulation parameters (coil orientation, intensity relative to motor threshold, and target muscle), and experimental designs. In addition, inconsistent reporting of variance measures and baseline normalization procedures precluded reliable pooling of effect sizes. Consequently, a structured narrative synthesis was deemed the most appropriate approach to summarize directionality, consistency, and physiological patterns across studies.

## 3 Results

### 3.1 Study Selection and Characteristics

The systematic search yielded 2936 records. After removal of 412 duplicates, 2524 titles and abstracts were screened for eligibility. Of these, 2472 were excluded for failing to meet inclusion criteria, leaving 52 full‐text articles for detailed assessment. After full‐text review, 17 studies met all eligibility criteria and were included in this review (Figure 1).

Of the 17 included studies, 11 directly examined upper‐limb–induced modulation of corticospinal or inhibitory parameters in lower‐limb muscles and therefore constitute the primary analytical focus of this review. The remaining studies investigated intersegmental or interhemispheric modulation in other muscle groups or neural circuits and were deliberately retained as secondary mechanistic evidence to support interpretation rather than as equivalent outcome studies.

All studies employed experimental or quasi‐experimental laboratory designs in healthy adults (total = 351 participants; sample sizes 9–63). A critical source of heterogeneity was the wide variation in experimental conditions, including contraction intensity (5%–75% MVC), contraction duration (brief vs. sustained), fatigue induction, and target muscles. Upper‐limb interventions consisted of voluntary or fatigue‐induced isometric contractions of hand, wrist, or elbow flexors, while outcomes were recorded from nonhomologous lower‐limb muscles (tibialis anterior, rectus femoris, soleus, or vastus lateralis) using TMS‐derived measures. These methodological differences are central to interpreting variability across findings rather than being treated as random noise. Table 1 summarizes study characteristics and key outcomes.

**TABLE 1:** Characteristics included studies examining intersegmental modulation between upper‐ and lower‐limb motor areas.

Author (year)	Sample (*n*)	Upper‐limb contraction protocol	TMS measures (lower limb)	Key findings/outcomes
Sasaki et al. 2020	14	Voluntary isometric elbow flexion (ipsilateral and contralateral)	MEPs, spinal reflexes (R LL)	↑ MEP amplitude during preparation/execution of elbow flexion.
Aboodarda et al. 2015	11	Elbow flexor fatigue (uni/bilateral)	VL TMS and M‐wave	+46% ↑ in corticospinal excitability postexercise (p = 0.003).
Chiou et al. 2013	15	Isometric ADM/FCR @ 75% max EMG (R side)	MEP, SICI (TA, RF)	↑ MEP amplitude and ↓ SICI during contraction (p < 0.001).
Matsuura et al. 2020	9	Handgrip 10% vs. 50% MVC, 120 s	MEP (VL)	10% MVC → ↑ MEP (+121%), no change at 50% MVC (*p* < 0.05).
Kato et al. 2022	22	Wrist flexion 10%–30% MVC, 1 min	MEP (SOL), SEP (P30–N70)	↑ MEP (*p* < 0.001); ↓ P50–N70 amplitude (*p* = 0.003).
Kato et al. 2025	19	Right wrist flexion @ 30% MVC + PAS	MEP, H‐reflex, SICI (SOL)	Synergistic ↑ MEP facilitation with PAS protocol.
Sasaki et al. 2021	12	Wrist flex/extension 0%–50% MVC	MEP (ES, RA, TA)	Task/intensity‐dependent ↑ corticospinal excitation.
Sasaki et al. 2018	11	Hand clenching	MEP (TA, RA, FDS)	Bilateral facilitation of lower limb MEPs during upper‐limb effort.
Zhou et al. 2017	14	Arm cycling	MEP (VL, TA)	↑ MEP in neurologically intact participants only.
Brownstein et al. 2021	12	Elbow flexors 20%/70% MVC, 4.5 min	TMS‐conditioned MEPs (BB, BR)	↓ conditioned MEPs (−33% @ 70% MVC; p < 0.01).
Paish et al. 2023	10	FDI maximal vs. submaximal	Silent period (FDI)	↑ SP duration +12.6% vs. 4.8% (p < 0.001).
Giovannelli et al. 2009	30	Max isometric FDI	iSP, CSP (TA and UL)	↑ iSP with contralateral contraction (p < 0.05).
Azevedo et al. 2022	13	BFO‐induced leg pain + exercise	MEP and SP (contra leg)	↑ SP and ↓ SICI (p < 0.05) → cross‐limb modulation.
Matsuya et al. 2017	16	FDI 5% MVC 15 s	MEP, SICI, Reciprocal Inhibition (SOL)	Cortical and spinal inhibition correlated (*r* = −0.56 to 0.66).
Rozand et al. 2019	63	FDI 10% MVC 2–3 s	MEP (VL, FDI)	↓ MEP with aging (1.76 → 1.15; p < 0.05); physical activity protective.
Fujiyama et al. 2012	30	Rhythmic vs. tonic ECR	SP (ECR)	Coordination/age modulate SP duration.
Tazoe et al. 2007	11	Tonic abductions (FDI/FCR/DEL)	MEP, CSP (various)	Remote contraction shortens CSP (intensity‐dependent).

Abbreviations: ADM, abductor digiti minimi; BB, biceps brachii; BFO, blood flow occlusion; BR, brachioradialis; CSP, cortical silent period; ECR, extensor carpi radialis; EMG, electromyography; ES, erector spinae; FCR, flexor carpi radialis; FDI, first dorsal interosseous; FDS, flexor digitorum superficialis; H‐reflex, Hoffmann reflex; ICF, intracortical facilitation; iSP, ipsilateral silent period; LAI, long‐latency afferent inhibition; LEP, laser‐evoked potential; LL, lower limb; MEP, motor evoked potential; MVC, maximal voluntary contraction; PAS, paired associative stimulation; RA, rectus abdominis; RF, rectus femoris; SAI, short‐latency afferent inhibition; SEP, somatosensory evoked potential; SICI, short‐interval intracortical inhibition; SOL, soleus; SP, silent period; TA, tibialis anterior; TMS, transcranial magnetic stimulation; UL, upper limb; VL, vastus lateralis.

### 3.2 Corticospinal Excitability

Fifteen of the included studies demonstrated significant facilitation of corticospinal excitability in lower‐limb muscles during or immediately after upper‐limb contraction. However, the strength, consistency, and direction of this facilitation depended strongly on experimental context. Among these, 11 studies directly assessed upper‐limb–to–lower‐limb modulation, representing the primary evidence base of this review (e.g., Chiou et al. 2013; Matsuura et al. 2020; Kato et al. 2022, 2025; Sasaki et al. 2018, 2020, 2021; Zhou et al. 2017; Azevedo et al. 2022; Matsuya et al. 2017).

The magnitude of facilitation ranged from +46% to +121%, with contraction intensity emerging as a key moderating variable, depending on contraction intensity and task demands. Chiou et al. (2013) and Aboodarda et al. (2015) reported marked increases in MEP amplitude in the tibialis anterior and vastus lateralis after high‐intensity or fatiguing contractions (*p* < 0.01). Matsuura et al. (2020) observed a 121% increase in MEPs at 10% MVC, with no further gain at higher intensity, suggesting an optimal submaximal range for facilitation, suggesting a nonlinear, saturating response rather than a monotonic intensity–excitability relationship. Kato et al. (2022, 2025) confirmed that voluntary wrist flexion (10%–30% MVC) and protocols combining paired associative stimulation (PAS) with isometric contraction enhanced excitability and H‐reflex amplitude in the soleus (*p* < 0.001), indicating convergence of supraspinal and spinal mechanisms under optimized conditions. Sasaki et al. (2018, 2020, 2021) demonstrated intensity‐dependent facilitation across arm–trunk interactions, with bilateral effects during elbow or wrist activation. Zhou et al. (2017) reported similar facilitation during arm cycling, suggesting that dynamic upper‐limb effort can also modulate lower‐limb corticospinal output.

Taken together, these findings indicate that facilitation is not ubiquitous but contingent on task structure, intensity, and neural state, underscoring the need for careful experimental stratification.

### 3.3 Intracortical Inhibition and Facilitation

Eight studies assessed intracortical inhibitory and facilitatory circuits using paired‐pulse TMS paradigms. Rather than uniform disinhibition, inhibitory modulation showed marked task dependence. Among those recording lower‐limb muscles, most reported reductions in SICI or changes in cortical silent period (CSP) during upper‐limb activation, indicating modulation of inhibitory control in remote motor representations.

In lower‐limb representations, several studies reported reduced SICI during upper‐limb activation, particularly during submaximal or pain‐modulated contractions. Chiou et al. (2013) and Azevedo et al. (2022) found significant reductions in SICI (*p* < 0.05) in lower‐limb areas during strong or pain‐modulated hand contractions. Paish et al. (2023) demonstrated a dose‐dependent increase in CSP duration (+12.6% at maximal contraction, *p* < 0.001), while Giovannelli et al. (2009) identified longer iSPs during contralateral isometric effort (*p* < 0.05). Tazoe et al. (2007) reported intensity‐dependent shortening of CSP during remote muscle contraction.

Studies assessing only upper‐limb muscles (e.g., Paish et al. 2023; Brownstein et al. 2021; Fujiyama et al. 2012) showed parallel inhibitory adjustments, providing a mechanistic context for interpreting the lower‐limb findings, supporting the interpretation that inhibitory modulation follows general network‐level principles rather than limb‐specific rules.

Overall, inhibitory control appears dynamically regulated, with facilitation dominating at submaximal intensities and compensatory inhibition emerging under high‐demand or fatigued states.

### 3.4 Interhemispheric and Spinal Modulation

Five studies provided evidence of interhemispheric and cortico–spinal coupling mechanisms that contribute to remote modulation. Only a subset recorded lower‐limb outcomes directly (e.g., Matsuya et al. (2017), while others assessed interhemispheric or upper‐limb parameters (Giovannelli et al. 2009; Fujiyama et al. 2012).

Matsuya et al. (2017) found significant correlations between SICI and reciprocal inhibition in the soleus (*r* = −0.56; *p* < 0.05). Giovannelli et al. (2009) reported enhanced iSP during unilateral activation, indicating adjustments in transcallosal inhibitory networks. Rozand et al. (2019) showed that MEP modulation is influenced by age and coordination, including effects observed in the vastus lateralis.

These mechanistic studies, although not all directly measure lower‐limb muscles, provide supporting evidence for the involvement of cortical and spinal inhibitory pathways, which likely contribute to the remote effects observed in the primary lower‐limb studies. Although indirect, these findings converge on the involvement of distributed cortical and spinal inhibitory networks that plausibly underpin the remote effects observed in primary lower‐limb studies.

### 3.5 Influence of Intensity and Fatigue

Across studies, contraction intensity and fatigue consistently shaped both the magnitude and direction of modulation. The direction and magnitude of intersegmental modulation were strongly dependent on contraction intensity and fatigue. Studies evaluating lower‐limb outcomes consistently showed that moderate‐intensity contractions (10%–30% MVC) produced the largest facilitation, whereas maximal or prolonged efforts often led to transient suppression of excitability.

Aboodarda et al. (2015) and Matsuura et al. (2020) demonstrated facilitation at submaximal intensities, with diminishing returns or inhibitory shifts at higher intensities. Fatigue protocols (e.g., Aboodarda et al. 2015; Lahouti et al. 2019) elicited initial facilitation followed by postfatigue reductions in excitability. Importantly, similar intensity‐dependent profiles were observed in mechanistic upper‐limb studies, reinforcing the generalizability of this regulatory pattern across segments.

### 3.6 Integration of Findings

Rather than simply demonstrating the presence of remote effects, the collective evidence indicates that upper‐limb isometric contractions modulate lower‐limb motor pathways in a context‐dependent and intensity‐sensitive manner. Across all studies, upper‐limb isometric contractions elicited remote modulation of motor pathways. Primary lower‐limb studies consistently demonstrated facilitation of corticospinal excitability (approximately 70%), while secondary mechanistic studies documented concurrent adjustments in intracortical, interhemispheric, and spinal inhibitory circuits.

This integrated interpretation highlights that intersegmental modulation reflects a flexible, network‐level regulation of motor output rather than a fixed excitatory phenomenon, providing a more rigorous synthesis of experimental conditions and mechanisms than descriptive summarization alone.

### 3.7 Risk of Bias

The methodological quality of the included studies was evaluated using the Cochrane Risk of Bias 2.0 tool. Overall, the studies demonstrated moderate methodological rigor. Randomization and allocation procedures were clearly reported in 12 of the 17 trials (70%), while the remaining studies used within‐subject crossover designs without a detailed description of sequence generation.

Blinding of participants and assessors was generally not feasible because of the experimental nature of the interventions; however, objective electrophysiological outcomes (e.g., MEP amplitude and CSP duration) minimized the potential for detection bias. Incomplete outcome data and selective reporting were infrequent, with more than 80% of studies providing complete datasets and transparent statistical procedures.

**FIGURE 2:**
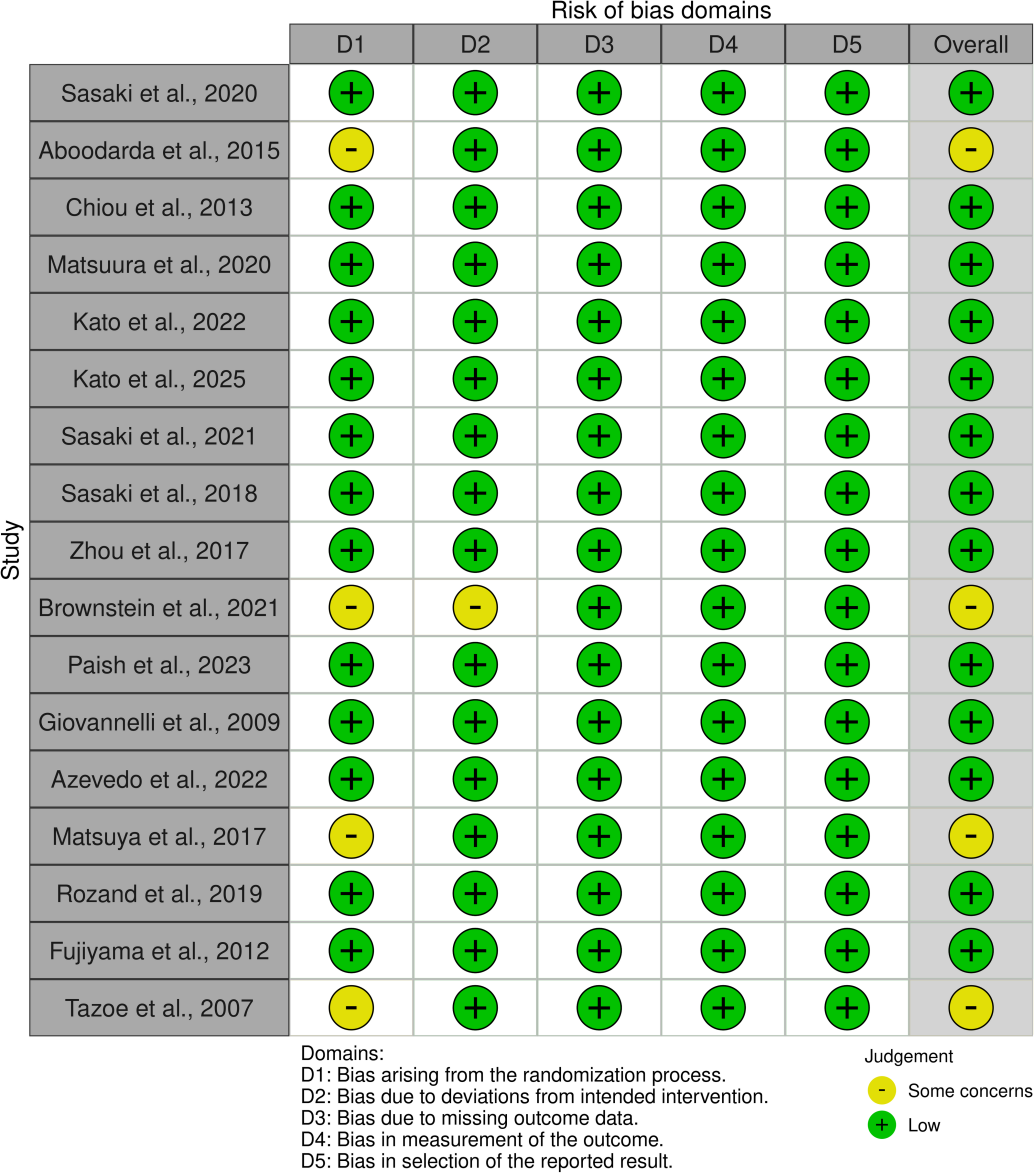
Risk of bias.

Variability in baseline control conditions (e.g., rest vs. light contraction) and inconsistent reporting of TMS parameters raised methodological concerns in nine studies. Based on aggregated RoB 2.0 ratings, six studies (35%) were judged to be at low risk of bias, eight (47%) presented some concerns, and three (18%) were rated as high risk. A visual summary of these assessments is provided in Figure 2.

## 4 Discussion

This systematic review synthesized experimental evidence examining how upper‐limb isometric contractions influence corticospinal excitability and inhibitory processes within lower‐limb motor representations. Across the included studies, a consistent pattern of remote facilitation emerged, particularly at moderate contraction intensities, whereas high‐intensity or fatiguing tasks tended to shift the system toward inhibitory dominance. These findings reinforce the concept that the human motor system operates through integrated supraspinal and spinal networks that dynamically redistribute excitability during voluntary effort.

A robust finding across the primary studies was a reduction in SICI during upper‐limb activation, indicating transient disinhibition of lower‐limb M1 circuits (Chiou et al. 2013; Azevedo et al. 2022). This observation directly relates to the seminal work by Kujirai et al. (1993), who first characterized SICI as a GABA‐A mediated intracortical inhibitory mechanism fundamental for regulating corticospinal output. Conversely, during maximal or sustained contractions, several studies reported prolonged CSP and increased iSP, suggesting enhanced interhemispheric inhibitory control (Paish et al. 2023). Classic evidence indicates that the iSP reflects transcallosal inhibitory processes that scale with task complexity and effort (Giovannelli et al. 2009). Together, these observations support a model in which facilitatory mechanisms dominate at moderate intensities, while inhibitory mechanisms emerge under high demand or fatigue, possibly to regulate excessive corticospinal drive.

Importantly, the intersegmental facilitation profile appears nonlinear. Studies evaluating lower‐limb outcomes consistently showed that 10%–30% MVC elicited the largest increases in MEP amplitude (Matsuura et al. 2020; Sasaki et al. 2021). Additional increases in intensity did not produce further facilitation, and maximal contractions often induced inhibitory rebound. Similar intensity‐dependent modulation has been described in mechanistic upper‐limb work (Škarabot et al. 2021), supporting the hypothesis that the motor network transitions from net facilitation to net inhibition once a physiological threshold is reached.

This nonlinear transition is consistent with principles of homeostatic metaplasticity, as described by the Bienenstock–Cooper–Munro (BCM) framework, whereby prior neuronal activity shifts the threshold for subsequent potentiation or depression. Experimental evidence indicates that exceeding intensity thresholds can trigger counter‐regulatory reductions in corticospinal excitability (Hassanzahraee et al. 2020a, 2020b). Fatigue protocols further supported this adaptive behavior; an initial excitatory phase followed by postfatigue suppression was observed (Aboodarda et al. 2015; Lahouti et al. 2019). In this context, Perez et al. (2012) proposed that task‐dependent modulation of facilitatory and inhibitory cortical circuits represents a core mechanism by which the motor system adapts to changing functional demands.

Although spinal circuits contribute to these effects, the collective evidence strongly indicates a predominantly supraspinal origin. Studies recording both cortical and spinal markers demonstrated that cortical changes often precede spinal adjustments and that remote contractions of small upper‐limb muscles (e.g., wrist or hand) can modify excitability of lower‐limb representations despite minimal proprioceptive influence on lumbosacral segments (Sasaki et al. 2018; Kato et al. 2022). These findings align with neuroimaging evidence showing that SMA, cingulate motor areas, and ipsilateral M1 are co‐recruited during unilateral actions, generating distributed modulatory signals that extend beyond the engaged limb. Classical physiological observations such as the Jendrassik maneuver further support the contribution of spinal circuits, as voluntary upper‐limb contraction enhances lower‐limb tendon reflex amplitude, indicating increased spinal reflex excitability. This convergence with H‐reflex findings reinforces that intersegmental modulation reflects coordinated supraspinal–spinal regulation (Zehr and Duysens 2004).

From a translational perspective, the consistent facilitation of lower‐limb corticospinal excitability observed during moderate‐intensity upper‐limb contractions suggests that remote voluntary effort may serve as a priming strategy for lower‐limb motor networks (Borzuola et al. 2024; Neva et al. 2021; Zehr and Duysens 2004). However, it is important to emphasize that all included studies were conducted in healthy adults under controlled laboratory conditions. Therefore, any clinical application in neurological populations remains speculative and requires direct empirical validation. Future studies should determine whether such intersegmental facilitation translates into meaningful functional gains when combined with gait training, balance tasks, or neuromodulatory interventions in clinical settings.

Despite the identified convergent patterns, the current evidence remains constrained by substantial methodological heterogeneity. Variability in contraction protocols, muscle selection, and timing of TMS measurements, and particularly in stimulation parameters such as coil orientation, hotspot determination, and input–output curves, limits comparability across studies (Rossini et al. 2015; Perez et al. 2012). Sample sizes were small across nearly all studies, reducing precision and increasing the risk of type II error. Additionally, most investigations focused narrowly on SICI, leaving other inhibitory circuits (SAI, LAI, RI) underexplored. The absence of studies using functional tasks, such as gait or postural control, further limits ecological interpretation.

Demographic moderators also warrant attention. Evidence suggests that age, physical activity, and coordination demands shape M1 excitability (Rozand et al. 2019; Fujiyama et al. 2012), yet these factors were inconsistently reported or controlled. Posture and coactivation, known to influence ipsilateral M1 engagement (Hendy et al. 2017), were also rarely standardized.

Overall, approximately 70% of the included studies demonstrated significant facilitation of lower‐limb corticospinal excitability, and about one‐third showed modulation of inhibitory or interhemispheric circuits. Together, these findings reinforce the interpretation that intersegmental modulation reflects a physiological expression of functional connectivity within the motor system, one that is intensity‐dependent, task‐specific, and shaped by coordinated cortical and spinal pathways.

Future work should prioritize standardized TMS protocols, adequately powered samples, and longitudinal or clinical studies to determine whether remote facilitation can meaningfully enhance rehabilitation outcomes. Examining underrepresented inhibitory pathways, dynamic tasks, and demographic moderators will be essential to refine mechanistic understanding and optimize clinical translation.

### 4.1 Limitations

The findings of this review should be interpreted in light of several methodological constraints inherent to the available literature. Although 17 experimental studies were included, most involved small samples (typically fewer than 20 participants) and heterogeneous task protocols, limiting generalizability. Differences in contraction type (voluntary versus fatigue‐induced), duration, and intensity (5%–75% MVC) hindered direct comparison and precluded quantitative meta‐analysis.

TMS methodologies also varied substantially across studies, including coil orientation, stimulation intensity, target muscle selection, and interstimulus intervals, introducing variability in measured MEPs and inhibitory parameters (SICI, ICF, and CSP). Only a subset of investigations systematically controlled for background EMG activity or coil positioning, factors known to influence reliability and reproducibility.

Finally, all included studies were conducted in healthy young adults under laboratory conditions. The absence of clinical populations, aging cohorts, or longitudinal training paradigms limits extrapolation to neurorehabilitation contexts and sustained neuroplastic adaptations. In line with established noninvasive brain stimulation guidelines (Rossini et al. 2015), insufficient standardization of stimulation parameters may substantially affect the interpretability of corticospinal and intracortical measures.

### 4.2 Clinical Implications and Future Directions

Although this review focused exclusively on healthy adults, the observed cross‐limb modulation of corticospinal excitability and inhibition has relevant implications for neurorehabilitation and motor training paradigms.

The consistent facilitation of lower‐limb excitability induced by high‐intensity or rhythmic upper‐limb contractions suggests that nonhomologous limb activation can enhance motor output and intersegmental coordination, potentially supporting recovery after stroke, spinal cord injury, or limb immobilization.

Integrating isometric upper‐limb contractions with neuromodulation techniques, such as rTMS, tDCS, or PAS, could optimize motor reorganization by exploiting natural interlimb connectivity. This approach may modulate task‐specific plasticity and facilitate rehabilitation when direct activation of target muscles is limited.

## 5 Conclusions

Taken together, the findings of this systematic review support the interpretation that intersegmental modulation reflects a physiological expression of distributed motor network connectivity rather than a nonspecific overflow phenomenon. This interpretation is consistent with classical frameworks of interlimb coordination, particularly the work of Zehr and Duysens (2004), which emphasizes the functional coupling between upper‐ and lower‐limb motor networks through shared neural circuitry. Upper‐limb isometric contractions induce intensity‐dependent and task‐specific adjustments in corticospinal excitability and inhibitory control of lower‐limb motor representations, primarily mediated by supraspinal mechanisms with contributory spinal involvement. These results highlight the integrative organization of the human motor system and provide a mechanistic framework for future experimental and translational investigations.

## Author Contributions

Gustavo Henrique de Mello Rosa conceptualized the study, conducted the literature search, performed data extraction, and drafted the manuscript. Gabrielly Santos Pereira and Marcelo Lourenço da Silva performed data extraction and methodological quality assessment. Fernanda da Silva Boni contributed to data interpretation and manuscript revision. João Eduardo de Araújo supervised the study, contributed to conceptual development, and critically revised the manuscript. All authors approved the final version of the manuscript.

## Funding

This work was supported by the Coordenação de Aperfeiçoamento de Pessoal de Nível Superior (No. 88887.817054/2023‐00).

## Conflicts of Interest

The authors declare no conflicts of interest.

## Supporting information


**Data S1:** Supporting Information.

## Data Availability

No data were used in the current manuscript.

## References

[ejn70501-bib-0001] Aboodarda, S. J. , D. B. Copithorne , K. E. Power , E. Drinkwater , and D. G. Behm . 2015. “Elbow Flexor Fatigue Modulates Central Excitability of the Knee Extensors.” Applied Physiology, Nutrition, and Metabolism 40: 924–930. 10.1139/apnm-2015-0088. 26300013

[ejn70501-bib-0002] Arya, K. N. , and S. Pandian . 2014. “Interlimb Neural Coupling: Implications for Poststroke Hemiparesis.” Annals of Physical and Rehabilitation Medicine 57: 696–713. 10.1016/j.rehab.2014.06.003. 25262645

[ejn70501-bib-0003] Azevedo, R. A. , D. Jazayeri , S. T. Yeung , et al. 2022. “The Effects of Pain Induced by Blood Flow Occlusion in One Leg on Exercise Tolerance and Corticospinal Excitability and Inhibition of the Contralateral Leg in Males.” Applied Physiology, Nutrition, and Metabolism 47: 632–648. 10.1139/apnm-2021-0597. 35201916

[ejn70501-bib-0004] Baldissera, F. , P. Borroni , P. Cavallari , and G. Cerri . 2002. “Excitability Changes in Human Corticospinal Projections to Forearm Muscles During Voluntary Movement of Ipsilateral Foot.” Journal of Physiology 539: 903–911. 10.1113/jphysiol.2001.013282. 11897859 PMC2290195

[ejn70501-bib-0005] Borzuola, R. , V. Caricati , M. Parrella , M. Scalia , and A. Macaluso . 2024. “Frequency‐Dependent Effects of Superimposed NMES on Spinal Excitability in Upper and Lower Limb Muscles.” Heliyon 10: e40145. 10.1016/j.heliyon.2024.e40145. 39568857 PMC11577215

[ejn70501-bib-0006] Brownstein, C. G. , L. Espeit , N. Royer , et al. 2021. “Reductions in Motoneuron Excitability During Sustained Isometric Contractions Are Dependent on Stimulus and Contraction Intensity.” Journal of Neurophysiology 125: 1636–1646. 10.1152/jn.00070.2021. 33788627

[ejn70501-bib-0007] Carroll, T. J. , R. D. Herbert , J. Munn , M. Lee , and S. C. Gandevia . 2006. “Contralateral Effects of Unilateral Strength Training: Evidence and Possible Mechanisms.” Journal of Applied Physiology 101: 1514–1522. 10.1152/japplphysiol.00531.2006. 17043329

[ejn70501-bib-0008] Chiou, S. Y. , R. Y. Wang , K. K. Liao , and Y. R. Yang . 2013. “Homologous Muscle Contraction During Unilateral Movement Does Not Show a Dominant Effect on Leg Representation of the Ipsilateral Primary Motor Cortex.” PLoS ONE 8: e72231. 10.1371/journal.pone.0072231. 23991067 PMC3749103

[ejn70501-bib-0009] Christensen, L. O. , P. Johannsen , T. Sinkjaer , N. Petersen , H. S. Pyndt , and J. B. Nielsen . 2000. “Cerebral Activation During Bicycle Movements in Man.” Experimental Brain Research 135: 66–72. 10.1007/s002210000493. 11104128

[ejn70501-bib-0010] Colomer‐Poveda, D. , S. Romero‐Arenas , M. Keller , T. Hortobágyi , and G. Márquez . 2019. “Effects of Acute and Chronic Unilateral Resistance Training Variables on Ipsilateral Motor Cortical Excitability and Cross‐Education: A Systematic Review.” Physical Therapy in Sport 40: 143–153. 10.1016/j.ptsp.2019.09.006. 31546134

[ejn70501-bib-0011] de Mello Rosa, G. H. , G. H. Moretto , K. Zhang , T. J. Chagas , and J. E. Araújo . 2025. “Modulation of Handgrip Strength by Contralateral Hip Flexor Motor Overflow: Randomized Controlled Trial.” Acta Fisiátrica 32: 88–94. 10.11606/issn.2317-0190.2024.236056.

[ejn70501-bib-0012] Debaere, F. , S. P. Swinnen , E. Béatse , S. Sunaert , P. Van Hecke , and J. Duysens . 2001. “Brain Areas Involved in Interlimb Coordination: A Distributed Network.” NeuroImage 14: 947–958. 10.1006/nimg.2001.0892. 11697927

[ejn70501-bib-0013] Dragert, K. , and E. P. Zehr . 2011. “Bilateral Neuromuscular Plasticity From Unilateral Training of the Ankle Dorsiflexors.” Experimental Brain Research 208: 217–227. 10.1007/s00221-010-2472-3. 21069308

[ejn70501-bib-0014] Ferbert, A. , A. Priori , J. C. Rothwell , B. L. Day , J. G. Colebatch , and C. D. Marsden . 1992. “Interhemispheric Inhibition of the Human Motor Cortex.” Journal of Physiology 453: 525–546. 10.1113/jphysiol.1992.sp019243. 1464843 PMC1175572

[ejn70501-bib-0015] Fujiyama, H. , M. R. Hinder , M. W. Schmidt , M. I. Garry , and J. J. Summers . 2012. “Age‐Related Differences in Corticospinal Excitability and Inhibition During Coordination of Upper and Lower Limbs.” Neurobiology of Aging 33: 1484.e1–1484.e14. 10.1016/j.neurobiolaging.2011.12.019. 22257984

[ejn70501-bib-0016] Giovannelli, F. , A. Borgheresi , F. Balestrieri , et al. 2009. “Modulation of Interhemispheric Inhibition by Volitional Motor Activity: An Ipsilateral Silent Period Study.” Journal of Physiology 587: 5393–5410. 10.1113/jphysiol.2009.175885. 19770195 PMC2793872

[ejn70501-bib-0017] Gordon, E. M. , R. J. Chauvin , A. N. Van , et al. 2023. “A Somato‐Cognitive Action Network Alternates With Effector Regions in Motor Cortex.” Nature 617: 351–359. 10.1038/s41586-023-05964-2. 37076628 PMC10172144

[ejn70501-bib-0018] Gueugneau, N. , S. Grosprêtre , P. Stapley , and R. Lepers . 2017. “High‐Frequency Neuromuscular Electrical Stimulation Modulates Interhemispheric Inhibition in Healthy Humans.” Journal of Neurophysiology 117: 467–475. 10.1152/jn.00355.2016. 27832594 PMC5263217

[ejn70501-bib-0019] Hanakawa, T. , I. Immisch , K. Toma , M. A. Dimyan , P. Van Gelderen , and M. Hallett . 2003. “Functional Properties of Brain Areas Associated With Motor Execution and Imagery.” Journal of Neurophysiology 89: 989–1002. 10.1152/jn.00132.2002. 12574475

[ejn70501-bib-0020] Hassanzahraee, M. , M. A. Nitsche , M. Zoghi , and S. Jaberzadeh . 2020a. “Determination of Anodal tDCS Duration Threshold for Reversal of Corticospinal Excitability: An Investigation for Induction of Counter‐Regulatory Mechanisms.” Brain Stimulation 13: 832–839. 10.1016/j.brs.2020.02.027. 32289714

[ejn70501-bib-0021] Hassanzahraee, M. , M. A. Nitsche , M. Zoghi , and S. Jaberzadeh . 2020b. “Determination of Anodal tDCS Intensity Threshold for Reversal of Corticospinal Excitability: An Investigation for Induction of Counter‐Regulatory Mechanisms.” Scientific Reports 10: 16108. 10.1038/s41598-020-72909-4. 32999375 PMC7527486

[ejn70501-bib-0022] Hendy, A. M. , S. Lamon , and D. J. Kidgell . 2017. “The Cross‐Education Phenomenon: Brain and Beyond.” Frontiers in Physiology 8: 297. 10.3389/fphys.2017.00297. 28539892 PMC5423908

[ejn70501-bib-0023] Hoy, K. E. , P. B. Fitzgerald , J. L. Bradshaw , C. A. Armatas , and N. Georgiou‐Karistianis . 2004. “Investigating the Cortical Origins of Motor Overflow.” Brain Research Reviews 46: 315–327. 10.1016/S0165-0173(04)00111-0. 15571773

[ejn70501-bib-0024] Kato, K. , T. Muraoka , N. Mizuguchi , K. Nakagawa , H. Nakata , and K. Kanosue . 2016. “Muscle Relaxation of the Foot Reduces Corticospinal Excitability of Hand Muscles and Enhances Intracortical Inhibition.” Frontiers in Human Neuroscience 10: 218. 10.3389/fnhum.2016.00218. 27242482 PMC4861736

[ejn70501-bib-0025] Kato, T. , N. Kaneko , and K. Nakazawa . 2025. “Stable Enhancement of Corticospinal Excitability by the Combination of Paired Associative Stimulation and Interlimb Cortical Network.” European Journal of Neuroscience 61: e70072. 10.1111/ejn.70072. 40197681 PMC11977449

[ejn70501-bib-0026] Kato, T. , N. Kaneko , A. Sasaki , et al. 2022. “Corticospinal Excitability and Somatosensory Information Processing of the Lower Limb Muscle During Upper Limb Voluntary or Electrically Induced Muscle Contractions.” European Journal of Neuroscience 55: 1810–1824. 10.1111/ejn.15643. 35274383

[ejn70501-bib-0027] Kujirai, T. , M. D. Caramia , J. C. Rothwell , et al. 1993. “Corticocortical Inhibition in Human Motor Cortex.” Journal of Physiology 471: 501–519. 10.1113/jphysiol.1993.sp019912. 8120818 PMC1143973

[ejn70501-bib-0028] Lahouti, B. , E. J. Lockyer , S. Wiseman , and D. C. Button . 2019. “Short‐Interval Intracortical Inhibition of the Biceps Brachii in Chronic‐Resistance Versus Non‐Resistance‐Trained Individuals.” Experimental Brain Research 237: 3023–3032. 10.1007/s00221-019-05649-1. 31529168

[ejn70501-bib-0029] Loadman, P. M. , and E. P. Zehr . 2007. “Rhythmic Arm Cycling Produces a Non‐Specific Signal That Suppresses Soleus H‐Reflex Amplitude in Stationary Legs.” Experimental Brain Research 179: 199–208. 10.1007/s00221-006-0782-2. 17119939

[ejn70501-bib-0030] Matsuura, R. , T. Yunoki , K. Shirakawa , and Y. Ohtsuka . 2020. “Effects of Sustained Unilateral Handgrip on Corticomotor Excitability in Both Knee Extensor Muscles.” European Journal of Applied Physiology 120: 1865–1879. 10.1007/s00421-020-04414-5. 32533244

[ejn70501-bib-0031] Matsuya, R. , J. Ushiyama , and J. Ushiba . 2017. “Inhibitory Interneuron Circuits at Cortical and Spinal Levels Are Associated With Individual Differences in Corticomuscular Coherence During Isometric Voluntary Contraction.” Scientific Reports 7: 44417. 10.1038/srep44417. 28290507 PMC5349562

[ejn70501-bib-0032] McIntyre‐Robinson, A. J. , and W. D. Byblow . 2013. “A Neurophysiological Basis for the Coordination Between Hand and Foot Movement.” Journal of Neurophysiology 110: 1039–1046. 10.1152/jn.00266.2013. 23741039

[ejn70501-bib-0034] Neva, J. , B. Greeley , B. Chau , et al. 2021. “Acute High‐Intensity Interval Exercise Modulates Corticospinal Excitability in Older Adults.” Medicine & Science in Sports & Exercise 54: 673–682. 10.1249/mss.0000000000002839. 34939609

[ejn70501-bib-0035] Paish, A. D. , A. M. Zero , C. J. McNeil , and C. L. Rice . 2023. “Increased Corticospinal Inhibition Following Brief Maximal and Submaximal Contractions in Humans.” Journal of Applied Physiology 135: 805–811. 10.1152/japplphysiol.00206.2023. 37616335

[ejn70501-bib-0036] Perez, M. A. , B. K. Lungholt , K. Nyborg , and J. B. Nielsen . 2004. “Motor Skill Training Induces Changes in the Excitability of the Leg Cortical Area in Healthy Humans.” Experimental Brain Research 159: 197–205. 10.1007/s00221-004-1947-5. 15549279

[ejn70501-bib-0037] Perez, M. A. , D. S. Soteropoulos , and S. N. Baker . 2012. “Corticomuscular Coherence During Bilateral Isometric Arm Voluntary Activity in Healthy Humans.” Journal of Neurophysiology 107: 2154–2162. 10.1152/jn.00722.2011. 22279195 PMC3331598

[ejn70501-bib-0038] Rossini, P. M. , D. Burke , R. Chen , et al. 2015. “Non‐Invasive Electrical and Magnetic Stimulation of the Brain, Spinal Cord, Roots and Peripheral Nerves: Basic Principles and Procedures for Routine Clinical and Research Application. An Updated Report From an I.F.C.N. Committee.” Clinical Neurophysiology 126: 1071–1107. 10.1016/j.clinph.2015.02.001. 25797650 PMC6350257

[ejn70501-bib-0039] Rozand, V. , J. W. Senefeld , C. W. Sundberg , A. E. Smith , and S. K. Hunter . 2019. “Differential Effects of Aging and Physical Activity on Corticospinal Excitability of Upper and Lower Limb Muscles.” Journal of Neurophysiology 122: 241–250. 10.1152/jn.00077.2019. 31091158 PMC6689774

[ejn70501-bib-0040] Sasaki, A. , N. Kaneko , Y. Masugi , T. Kato , M. Milosevic , and K. Nakazawa . 2021. “Task‐ and Intensity‐Dependent Modulation of Arm‐Trunk Neural Interactions in the Corticospinal Pathway in Humans.” eNeuro 8: ENEURO.0111‐21.2021. 10.1523/ENEURO.0111-21.2021. PMC8482852 34503966

[ejn70501-bib-0041] Sasaki, A. , N. Kaneko , Y. Masugi , M. Milosevic , and K. Nakazawa . 2020. “Interlimb Neural Interactions in Corticospinal and Spinal Reflex Circuits During Preparation and Execution of Isometric Elbow Flexion.” Journal of Neurophysiology 124: 652–667. 10.1152/jn.00705.2019. 32697605

[ejn70501-bib-0042] Sasaki, A. , M. Milosevic , H. Sekiguchi , and K. Nakazawa . 2018. “Evidence for Existence of Trunk‐Limb Neural Interaction in the Corticospinal Pathway.” Neuroscience Letters 668: 31–36. 10.1016/j.neulet.2018.01.011. 29309857

[ejn70501-bib-0043] Škarabot, J. , C. G. Brownstein , A. Casolo , A. Del Vecchio , and P. Ansdell . 2021. “The Knowns and Unknowns of Neural Adaptations to Resistance Training.” European Journal of Applied Physiology 121: 675–685. 10.1007/s00421-020-04567-3. 33355714 PMC7892509

[ejn70501-bib-0044] Sohn, Y. H. , and M. Hallett . 2004. “Surround Inhibition in Human Motor System.” Experimental Brain Research 158: 397–404. 10.1007/s00221-004-1909-y. 15146307

[ejn70501-bib-0045] Stinear, C. M. 2010. “Prediction of Recovery of Motor Function After Stroke.” Lancet Neurology 9: 1228–1232. 10.1016/S1474-4422(10)70247-7. 21035399

[ejn70501-bib-0046] Stinear, C. M. , and W. D. Byblow . 2004. “Modulation of Corticospinal Excitability and Intracortical Inhibition During Motor Imagery Is Task‐Dependent.” Experimental Brain Research 157: 351–358. 10.1007/s00221-004-1851-z. 14997259

[ejn70501-bib-0047] Swinnen, S. P. 2002. “Intermanual Coordination: From Behavioural Principles to Neural‐Network Interactions.” Nature Reviews Neuroscience 3: 348–359. 10.1038/nrn807. 11988774

[ejn70501-bib-0048] Swinnen, S. P. , and R. G. Carson . 2002. “The Control and Learning of Patterns of Interlimb Coordination: Past and Present Issues in Normal and Disordered Control.” Acta Psychologica 110: 129–137. 10.1016/S0001-6918(02)00030-6. 12102102

[ejn70501-bib-0049] Tazoe, T. , and T. Komiyama . 2014. “Interlimb Neural Interactions in the Corticospinal Pathways.” Journal of Physical Fitness and Sports Medicine 3: 181–190. 10.7600/jpfsm.3.181.

[ejn70501-bib-0050] Tazoe, T. , M. Sakamoto , T. Nakajima , T. Endoh , and T. Komiyama . 2007. “Effects of Remote Muscle Contraction on Transcranial Magnetic Stimulation‐Induced Motor Evoked Potentials and Silent Periods in Humans.” Clinical Neurophysiology 118: 1204–1212. 10.1016/j.clinph.2007.03.005. 17449319

[ejn70501-bib-0051] Zehr, E. P. , and J. Duysens . 2004. “Regulation of Arm and Leg Movement During Human Locomotion.” Neuroscientist 10: 347–361. 10.1177/1073858404264680. 15271262

[ejn70501-bib-0052] Zhou, R. , L. Alvarado , S. Kim , S. L. Chong , and V. K. Mushahwar . 2017. “Modulation of Corticospinal Input to the Legs by Arm and Leg Cycling in People With Incomplete Spinal Cord Injury.” Journal of Neurophysiology 118: 2507–2519. 10.1152/jn.00663.2016. 28701544 PMC5646203

