# Supplementary material for: Intersegmental Modulation of Lower‐Limb Corticospinal Excitability and Inhibition Induced by Upper‐Limb Isometric Contractions: A Systematic Review
Source: Eur J Neurosci. 2026 Apr 12;63(7):e70501. doi: 10.1111/ejn.70501 (PMC13071246; doi:10.1111/ejn.70501)
Supplement: Supplementary file 1 — Data S1: Supporting Information. [file EJN-63-0-s001.docx]

**Supplementary Appendix 1**

Full Electronic Search Strategies

This appendix provides the complete database-specific search strategies used in this systematic review. Searches were conducted between January 2007 and November 2025. No language restrictions were applied. Filters for humans and adults (18–65 years) were applied when available.

1. PubMed/MEDLINE

(("Transcranial Magnetic Stimulation"[MeSH] OR "transcranial magnetic stimulation" OR TMS OR
"motor evoked potential*" OR MEP OR "corticospinal excitability" OR "intracortical inhibition"
OR "intracortical facilitation" OR SICI OR ICF OR "ipsilateral silent period" OR iSP
OR "cortical silent period" OR CSP))
AND
(("Isometric Contraction"[MeSH] OR "isometric contraction*" OR handgrip OR "upper limb"
OR "upper extremity" OR arm OR "wrist flexion" OR "elbow flexion" OR "shoulder contraction"))
AND
(("Lower Extremity"[MeSH] OR "lower limb" OR "lower extremity" OR leg
OR "tibialis anterior" OR soleus OR "vastus lateralis" OR quadriceps))
AND
(("intersegmental modulation" OR intersegmental OR interlimb OR
"interlimb interaction" OR "cross-limb" OR "cross education" OR "remote muscle activation"))

2. EMBASE

('transcranial magnetic stimulation'/exp OR 'motor evoked potential' OR
'corticospinal excitability' OR 'intracortical inhibition' OR 'intracortical facilitation'
OR sici OR icf OR 'ipsilateral silent period' OR 'cortical silent period')
AND
('isometric contraction'/exp OR handgrip OR 'upper limb' OR 'upper extremity'
OR 'wrist flexion' OR 'elbow flexion' OR 'shoulder contraction')
AND
('lower limb'/exp OR 'lower extremity' OR 'tibialis anterior'
OR soleus OR 'vastus lateralis' OR quadriceps)
AND
('intersegmental modulation' OR intersegmental OR interlimb
OR 'cross education' OR 'remote activation')

3. Scopus

TITLE-ABS-KEY ("transcranial magnetic stimulation" OR TMS OR "motor evoked potential*"
OR "corticospinal excitability" OR SICI OR ICF OR "silent period")
AND
TITLE-ABS-KEY ("isometric contraction" OR handgrip OR "upper limb"
OR "wrist flexion" OR "elbow flexion")
AND
TITLE-ABS-KEY ("lower limb" OR leg OR "tibialis anterior"
OR soleus OR "vastus lateralis")
AND
TITLE-ABS-KEY ("intersegmental" OR "interlimb" OR
"cross-limb" OR "remote activation")

4. Web of Science

TS=("transcranial magnetic stimulation" OR TMS OR "motor evoked potential*"
OR "corticospinal excitability" OR SICI OR ICF OR "silent period")
AND
TS=("isometric contraction" OR handgrip OR "upper limb"
OR "wrist flexion" OR "elbow flexion")
AND
TS=("lower limb" OR leg OR "tibialis anterior"
OR soleus OR "vastus lateralis")
AND
TS=("intersegmental" OR "interlimb interaction"
OR "cross education" OR "remote muscle")

5. LILACS

("estimulação magnética transcraniana" OR "transcranial magnetic stimulation"
OR "potencial evocado motor")
AND
("contração isométrica" OR "handgrip" OR "membro superior")
AND
("membro inferior" OR "tibial anterior" OR "sóleo")
AND
("intersegmentar" OR "intermembro" OR "ativação remota")
